# Tea and its components reduce the production of uric acid by inhibiting xanthine oxidase

**DOI:** 10.29219/fnr.v66.8239

**Published:** 2022-06-15

**Authors:** Dan Wu, Ruohong Chen, Wenji Zhang, Xingfei Lai, Lingli Sun, Qiuhua Li, Zhenbiao Zhang, Junxi Cao, Shuai Wen, Zhaoxiang Lai, Zhigang Li, Fanrong Cao, Shili Sun

**Affiliations:** 1 Tea Research Institute, Guangdong Academy of Agricultural Sciences/Guangdong Provincial Key Laboratory of Tea Plant Resources Innovation & Utilization, Guangzhou, China; 2 College of Horticulture, South China Agricultural University, Guangzhou, China

**Keywords:** tea, gallic acid, tea polyphenols, xanthine oxidase, uric acid, hyperuricemia

## Abstract

**Background:**

The health benefits of tea are as diverse including the reduction of uric acid levels. Xanthine oxidase is the most directly mediated enzyme in the production of uric acid.

**Objective:**

To explore the inhibitory effects of different teas and its main bioactive components on the production of uric acid.

**Design:**

Experimental study. The experiments were conducted in vitro using human immortalized normal liver cell line HL-7702 (L-02).

**Results:**

The inhibition of the xanthine oxidase activities and the expression level of xanthine dehydrogenase mRNA stimulated in the hyperuric hepatocyte cell model showed that the unfermented green tea and th1e lightly fermented yellow tea, white tea, and oolong tea significantly stronger than the highly fermented black tea and dark tea. The main bioactive compound, gallic acid, showed the strongest inhibitory effect on uric acid production, followed by tea polyphenols and theaflavins.

**Discussion:**

All teas exhibited significant inhibition of xanthine oxidase activities, and the degree of fermentation of tea may be inversely proportional to its ability to inhibit the production of uric acid. Compared with tea polyphenols rich in tea, gallic acid may be a more potential uric acid-lowering component.

**Conclusion:**

In this article, we first compared the effects of six traditional Chinese tea made from a single variety in stabilizing the synthesis of uric acid and found that the lighter the fermentation, the greater the potential for inhibiting the production of uric acid. Furthermore, we analyzed the inhibitory effects of its main biochemical active ingredients and found that the inhibitory effects of polyphenols rich in lightly fermented tea were significantly stronger than caffeine rich in highly fermented tea. Our findings will be helpful for people to choose a proper tea for alleviating hyperuricemia and provide a scientific basis for uric acid-lowering tea processing.

## Graphical abstract

**Figure:**
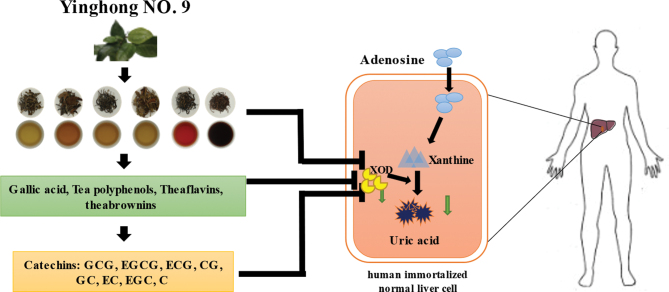

## Popular scientific summary

- Using human normal liver cells, a hyperuricemia model was established.
- Comparison of various teas and its constituents in inhibiting the uric acid production.
- The degree of fermentation determines the XOD activity.
- Gallic acid significantly inhibits the production of uric acid at the cellular level.
- Ester catechins had a stronger effect than non-ester catechins.

Tea (*Camellia sinensis*), originated in China, is presently the second most consumed beverage worldwide after water, with an estimated two billion cups every day (1). It has multiple health benefits that are largely attributed to the bioactive compounds, such as polyphenols, theaflavins, free amino acids, purine alkaloids, etc. (2), which have cardioprotective, neuroprotection, anti-inflammatory, antioxidation, and anti-hyperuric acid effects (3). Traditional Chinese tea is divided into six types – green tea, white tea, yellow tea, oolong tea, black tea, and dark tea – on the basis of processing methods. Green tea is prepared by de-enzyming, rolling, and drying. White tea is the simplest type of tea and prepared by withering and drying fresh tea leaves. An additional yellowing step is added between rolling and drying green tea leaves to make yellow tea, whereas withering and rocking green tea leaves before enzyme removal results in the highly aromatic oolong tea. Black tea is a completely fermented tea that undergoes withering, rolling or rolling cutting, fermentation, and drying. Compared with green tea, dark tea is piling fermented and dried after rolling and is a type of tea with a unique fermentation method by microbial fermentation (4). Based on these processing steps, tea is also classified as non-fermented (green), lightly fermented (white, yellow, and oolong), and heavily fermented (black and dark) (5). Different processing methods endow tea with distinct taste, aroma, and color and also affect the proportion of active compounds and consequently the biological effects (6). For instance, the inactivation of oxidoreductase during the preparation of green tea retains high levels of polyphenols with potent uric acid lowering, antioxidant, and anti-inflammatory effects (3). In contrast, fermentation is a form of oxidation that alters the composition of bioactive substances depending on the degree of fermentation. For instance, catechins often combine during fermentation to form complex substances, such as theaflavins, thearubigins, and other flavonoids, which endow the fermented tea with liquid lowering and anti-obesity properties. Wang et al. reported that oxidized tea polyphenols like theaflavins in fermented tea can regulate lipid metabolism (5).

Uric acid is the final product of human purine metabolism. Excess serum urate concentrations will reduce the survival rate of human (7). Therefore, maintaining the balance between uric acid synthesis and excretion is an important element of human health. Around 4/5th of the uric acid is produced in the liver by endogenous purine metabolism, and the remaining is the result of consuming foods rich in purines. Furthermore, two-thirds of uric acid are excreted through the kidney, and the rest through the intestine. In the event of kidney failure, the intestine becomes the primary route of uric acid excretion (8). Hyperuricemia is defined an increase in serum uric acid concentration above the saturation threshold, which is 7 mg/dL (400 μM) in humans (9). It is the direct result of aberrant uric acid metabolism and can progress to gout, metabolic syndrome, hypertension, cardiovascular disease, and obesity (10). Uric acid is synthesized by xanthine oxidase (XOD; encoded by the XDH gene), which oxidizes hypoxanthine to xanthine and the latter to uric acid (11). Although XOD inhibitors can reduce uric acid synthesis, they may increase the morbidity and mortality of cardiovascular diseases (12). Therefore, it is essential to discover natural compounds that can regulate the metabolism of uric acid with lower side effects.

Tea had been documented for uric acid-lowing properties, which are largely attributed to tea polyphenols (mainly EGCG in catechins), theaflavins (theaflavin, theaflavin-3-gallate, and theaflavin-3,3’-digallate) (13), and gallic acid (14). In this study, we compared the uric acid-lowering effects of six different types of tea from the same species as well as that of the key bioactive compounds, by analyzing XOD enzyme activity. Our research will be better and more accurately guide the actual production and processing of novel tea with special effects of lowering uric acid. Furthermore, it can provide the foundation for future development of related uric acid-lowering special health food to help patients with hyperuricemia.

## Materials and methods

### Chemicals and reagents

Seventeen samples of the Yinghong No. 9 tea cultivar (six types of tea, black tea fermented for 0, 2, 5, and 8 h, and dark tea stacked for 0, 10, 20, 30, 40, 50, and 60 days) were obtained from the Tea Research Institute of Guangdong Academy of Agricultural Sciences, China. Tea components including C ((+)-Catechin, B21722, high performance liquid chromatography (HPLC) purity ≥ 98%), GC ((-)-Gallocatechin, B20849, HPLC ≥ 98%), EC (Epicatechin, B20102, HPLC ≥ 98%), EGC ((-)-Epigallocatechin, B20105, HPLC ≥ 98%), CG (Catechin gallate, B20350, HPLC ≥ 98%), ECG ((-)-Epicatechin gallate, B20103, HPLC ≥ 98%), GCG ((-)-Gallocatechin gallate, B20850, HPLC ≥ 98%), EGCG (Epigallocatechin gallate, B20106, HPLC ≥ 98%), and gallic acid (B20851, HPLC ≥ 98%) were purchased from Yuanye Biological technology (Shanghai, China). Tea polyphenols (HPLC > 98%), tea polysaccharides (HPLC ≥ 80%), and theaflavins (HPLC ≥ 90%) were extracted from Tea Research Institute of Guangdong Academy of Agricultural Sciences, China. L-L-theanine (C7H14N203, 3081-61-6, HPLC ≥ 98%) was purchased from USP Reference Standard (USA), and theabrownins (2020TB0717, HPLC > 90%) was purchased from Hangzhou Qinyuan Natural Plant High-Tech Co. Ltd (Hangzhou, Zhejiang, China). All reagents were stored at 2–8°C. Peroxidase (POD) (S10062, HPLC ≥ 98%), allopurinol (B27249, HPLC ≥ 98%), 2,6-dihydroxypurine (B20561, HPLC ≥ 98%), 4-aminoantipyrine (B34212, HPLC ≥ 98%), XOD (S10113, HPLC ≥ 98%), and (-)-epicatechin gallate (Cat#B20103, HPLC ≥ 98%) were purchased from Yuanye Biological Technology (Shanghai, China) and stored at −20°C. XOD (X1875, Sigma, USA) and caffeine (P/N: 71559, HPLC = 99.8%, TMstandard, Beijing, China) were also stored at −20°C. Ethyl acetate (w/% ≥ 99.5%), 95% ethanol (w/% ≥ 95%), n-butanol (w/% ≥ 99.5%) were purchased from Tianjin Yongda Chemical Reagent Co., Ltd and stored at room temperature.

### Sample preparation

**Fig. 1:**
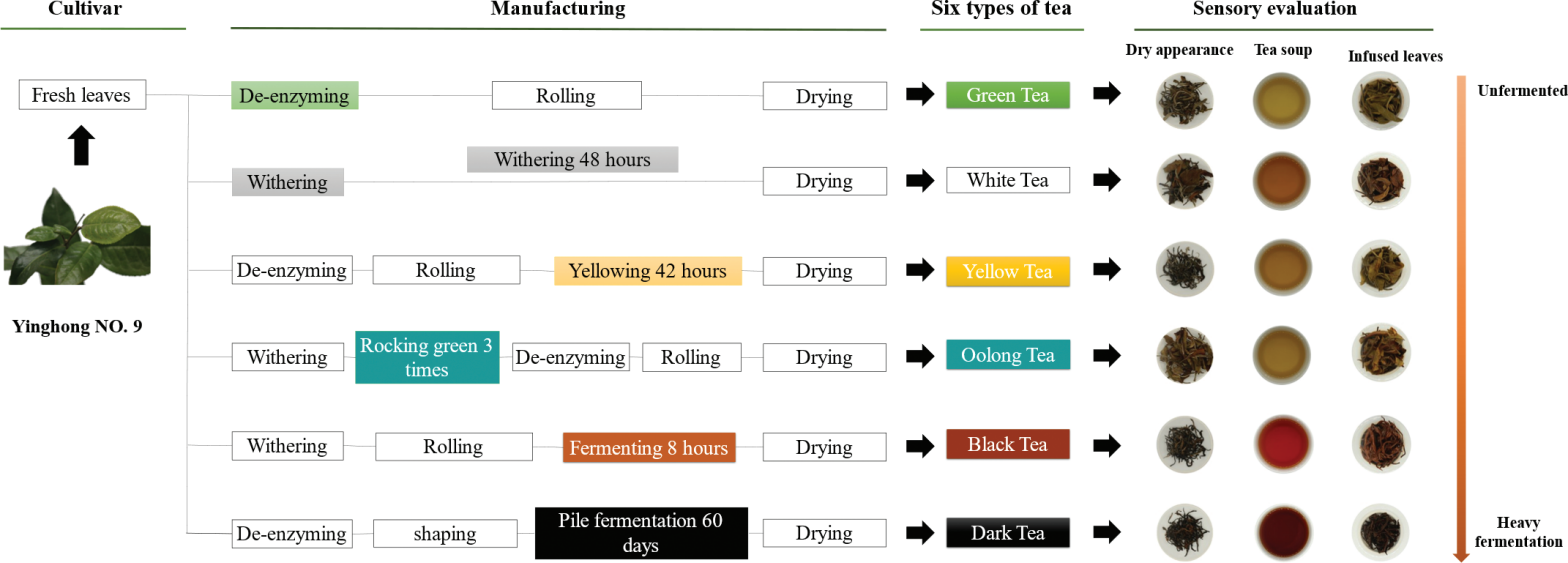
Traditional manufacturing processes of six types of tea. The picture depicts Yinghong No. 9 tea varieties from fresh leaves to manufacturing to finished tea and finally to sensory evaluation; in the description of the manufacturing process, the representative of the manufacturing process with the same color as each type of tea is the key processing process for that type of tea. ‘Dry appearance’ represents the shape and color of the tea before brewing; ‘Tea soup’ represents the color of the tea after brewing the tea, and ‘Infused leaves’ represents the shape and color of the tea after brewing. The direction of the arrow indicates the degree of fermentation.

All tea samples were processed in Tea Research Institute of Guangdong Academy of Agricultural Sciences using fresh leaves of a uniform specification of one bud with two to six leaves in 2020. Briefly, fresh tea leaves were heated at 230°C to inactivate the endogenous enzymes, rolled for 30 min and then directly dried into green tea. White tea was prepared by withering the fresh tea leaves at room temperature for 48 h and then dried. Another part of the fresh leaves was fixed at 180°C to inactivate most of the enzymes, rolled for 30 min, yellowed at room temperature and 70% relative humidity for 42 h, and finally dried into yellow tea. Oolong tea is the most aromatic type of tea. Tea leaves were rocking green three times after withering, then fixed at 230°C, rolled for 30 min, and finally dried into oolong tea. Black tea was prepared by withering the fresh tea leaves for 6–8 h, rolled for 30 min, fermentation with endogenous enzymes at room temperature and 90% relative humidity for 8 h, and drying. It is a type of fully fermented tea. To prepare dark tea, the tea leaves were withered and heated at 230°C to inactivate the enzymes, rolled for 30 min, and then pile-fermented for 60 days. The pile was turned over, the temperature was adjusted as required during the fermentation period, and the fermented leaves were dried (Fig. 1). According to the processing methods of black tea, we set the fermentation time to four gradients of 0, 2, 5, and 8 h to make black tea with different degrees of fermentation. Similarly, we set the stack fermentation time to a gradient of 0, 10, 20, 30, 40, 50, and 60 days to make dark tea with different stacking times.

The sensory evaluation of samples was evaluated according to the Chinese national standard procedure (GB/T 23776, 2018) (15): briefly, 3 g of tea leaves was steeped in 110 mL freshly boiled water for 5 min (of note, green tea was brewed for 4 min and dark tea for 2 min, after which the tea soup was poured out and brewed again for 5 min). Then, we can get the tea soup, and the tea residue was filtered out after brewing the infused leaves.

Seventeen samples of tea were extracted three times using boiled distilled water (tea/water (w/v) = 1:20) for 30 min. The extracts were filtered, frozen overnight at −80°C, and lyophilized in a freeze dryer (FD-1A-50, Biocool) for 24 h. The lyophilized powders were collected in moisture-proof bags and desiccated before storage.

### Determination of XOD inhibitory activity in vitro

XOD activity was evaluated by the double enzyme coupling method as described by He et al. (16) with slight modifications. The chromogenic solution was first prepared by diluting 0.0619 g 4-aminoantipyrine, 0.006 g horseradish peroxidase, and 0.168 g phenol in 300 mL, 0.05 mol/L Tris-HCl buffer pH 8.0, and stored at 4°C. For the reaction, 0.2 mL of sample solution (the action concentration of tea, tea components, and positive drug (allopurinol) are 2, 0.12, and 0.064 mg/mL, respectively) and 0.05 mL of 0.52 U/mL XOD solution were mixed and incubated at 37°C for 10 min, followed by the addition of 0.4 mL 0.22 mmol/L xanthine solution and 3.05 mL chromogenic solution. The mixture was incubated at 37°C for 20 min, and the reaction was terminated by inactivating the enzyme with 0.1 mL 1 mol/L NaOH solution. After cooling to room temperature, the absorbance was measured at 508 nm, and the system without extract samples and enzymes was used as a blank to zero.


$$\text{XOD inhibitory rate }(\%)=\frac{\left(A_{0}-\left(A_{2}-A_{1}\right)\right)}{A_{0}}\times 100\%$$

where A_0_ is the sample blank system without xanthine, A_2_ is the sample group, and A_1_ is the positive blank system without extract samples.

### Biochemical assays

Tea polyphenols were measured by the Folinphenol method (17) and free amino acids by ninhydrincolourimetry (18), and the anthrone–sulfuric acid colorimetric assay was used to measure the total soluble sugar content (19). Besides, theaflavins, thearubigins, and theabrownins were measured according to the People’s Republic of China Agricultural Industry Standard (NY/T 3675-2020). Caffeine, gallic acid, and catechin levels were measured by high-performance liquid chromatography (HPLC) (20). The correlation analysis between the biochemical contents and the inhibitory activity of tea on XOD activity was calculated using Excel 2016.

### Cell culture

The human immortalized normal liver cell line HL-7702 (L-02) was maintained in RPMI 1640 (Corning, New York State, USA) supplemented with 10% (v/v) fetal bovine serum, penicillin (0.1 mg/mL), and streptomycin (0.1 mg/mL) (Thermo Fisher Scientific, Waltham, Massachusetts, USA). The cells were cultured in a humidified incubator at 37°C and 5% CO_2_.

### MTT assay

Cell viability was measured using 3-(4,5-dimethylthiazolyl-2)-2,5-diphenyltetrazolium bromide (MTT) as described previously (21). The L-02 cells were seeded in a 96-well plate at the density of 1 × 10^5^ cells/well and treated with different concentrations of adenosine for 24 h. MTT was then added into each well at the final concentration of 0.5 mg/mL, and the cells were incubated for 4 h. After dissolving formazan crystals with 0.15 mL DMSO, the optical density was measured at 570 nm on a plate reader (Berthold Technologies).

### Cellular hyperuricemia model

L-02 cells are plated in 24-well plates at the density of 2.5 × 10^5^ cells/well and cultured overnight. Following 24 h induction with 1 mM adenosine in serum-free medium, 0.005 U/mL XOD was added, and the cells were incubated for 8 h. All incubation processes are carried out at 37°C and 5% CO_2_ in a humidified incubator.

### Uric acid measurement

The L-02 cells were induced with 1 mM adenosine as described above and then incubated with different tea samples for 2 h before incubating with 0.005 U/mL XOD for 8 h, and the uric acid content in the cell culture supernatant was measured using a uric acid test kit according to the manufacturer’s instructions (C012-2-1, Nanjing Jiancheng Bioengineering Institute, China).

### Cellular XOD activity measurement

XOD activity of hyperuricemia hepatocytes was measured using the colorimetric XOD assay kit according to the manufacturer’s instructions (A002-1-1, Nanjing Jiancheng Bioengineering Institute, China).

### Total RNA isolation and real-time quantitative PCR

Total RNA was isolated from L-02 cells using the Total RNA Kit I (Omega Bio-Tek, Guangzhou, China), and then the first strand cDNA was synthesized using a reverse transcription kit (TOYOBO Co., Ltd, Osaka, Japan). Real-time quantitative polymerase chain reaction (RT-qPCR) was performed on the ABI7500 Real-Time System with the SYBR Green PCR Master Mix (YEASEN, Shanghai, China). A three-step amplification program was used: pre-denaturation at 95°C for 5 min, 40 cycles of 95°C for 10 s, 60°C for 20 s, and 72°C for 20 s. The primers sequences are as follows: XDH – 5´-GGACAGTTGTGGCTCTTGAGGT-3´and 5´-GGAAGGTTGGTTTTGCACAGCC-3´; β-actin – 5´-CACCATTGGCAATGAGCGGTTC-3´ and 5´-AGGTCTTTGCGGATGTCCACGT-3´. The relative gene expression levels were normalized to that of β-actin.

### Statistical analysis

Data are expressed as the mean ± standard deviation of at least three independent experiments. GraphPad Prism 8.0 was used for data analysis. Analysis of variance (ANOVA) and Tukey’s post-hoc tests were used to compare multiple groups.

## Results

### Tea limits uric acid production by inhibiting XOD activity in vitro

**Fig. 2:**
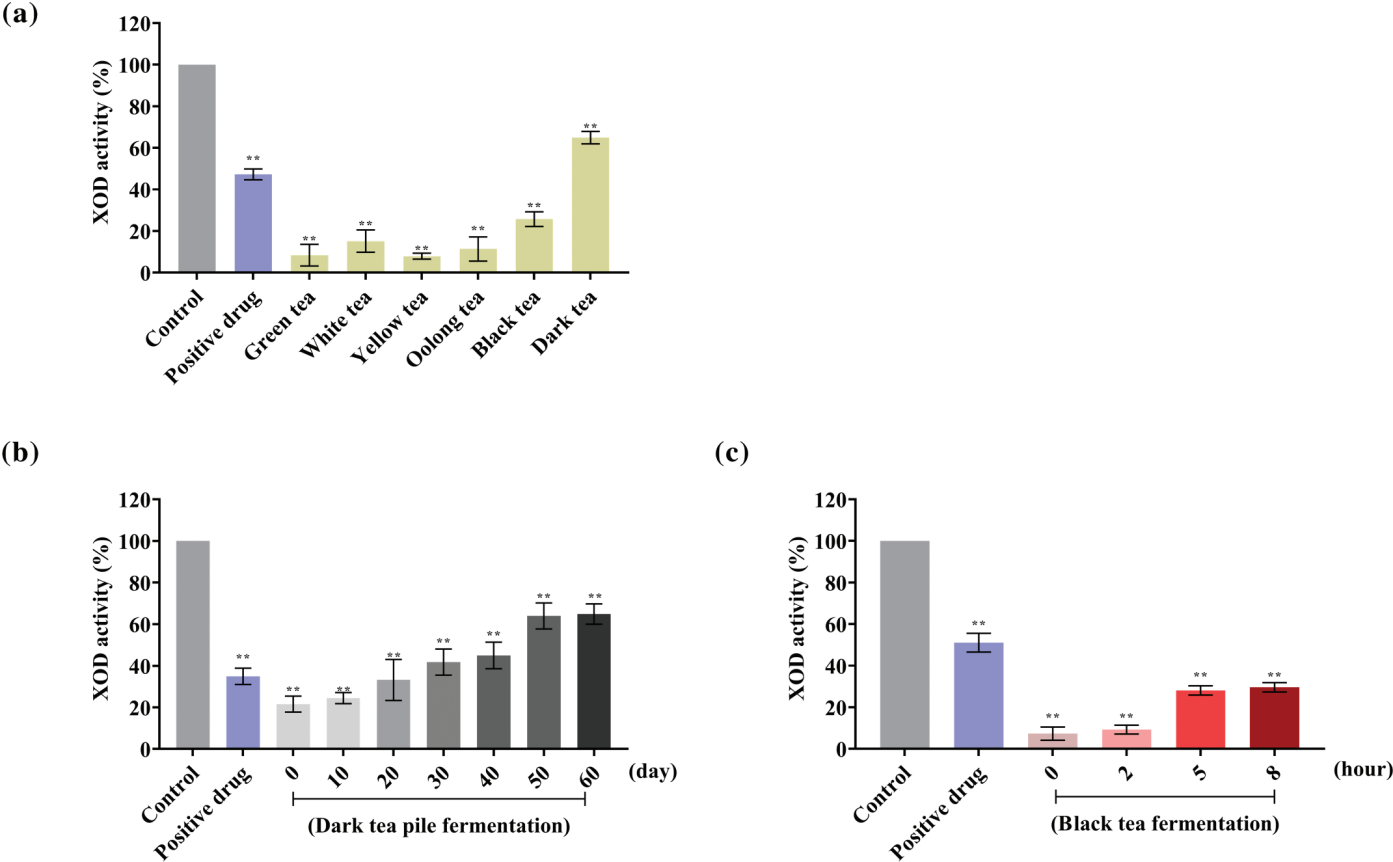
XOD activity of tea. (a) XOD activity in the presence of different teas; (b) XOD activity in the presence of dark tea fermented for 0, 10, 20, 30, 40, 50, and 60 days. (c) XOD activity in the presence of black tea fermented for 0, 2, 5, and 8 h. The data are expressed as mean ± SD. ^ns^*P* > 0.1, *P < 0.1, **P < 0.01, ***P < 0.001, and ****P < 0.0001 are compared with the control.

To assess the XOD inhibition activities of the samples, the double enzyme coupling method was performed. As shown in Fig. 2a, the green tea had the strongest inhibitory effect, followed by yellow tea, oolong tea, white tea, black tea, and dark tea, which indicated a negative correlation between the degree of fermentation and XOD inhibition. To validate this hypothesis, we, furthermore, compared the XOD inhibitory effect of black tea and dark tea samples fermented for varying duration and found that the inhibitory effect of tea weakened with longer fermentation (Fig. 2b, c).

### Correlation between uric acid reduction and bioactive compounds

**Fig. 3:**
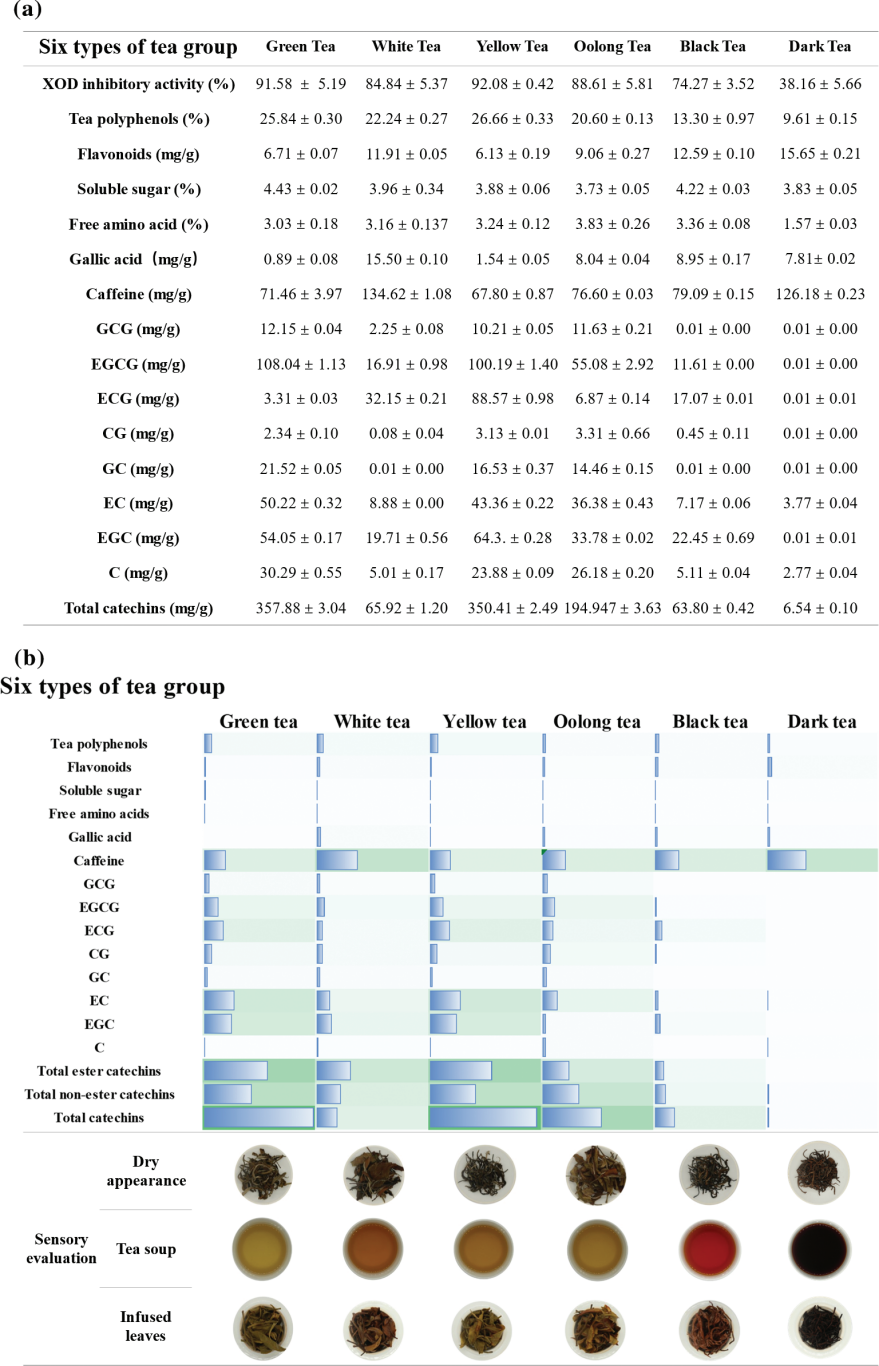
Biochemical components and sensory evaluation of six major teas. (a) The content of biochemical components in six types of tea. (b) 1) Heat map showing changes in biochemical components and sensory evaluation. The length of the rectangle and the color intensity indicate the content. 2) The results of sensory evaluation in six types of tea. As the degree of fermentation deepens, the color of the dry tea, the brewed tea soup, and the brewed tea leaves were gradually deepen.

**Fig. 4:**
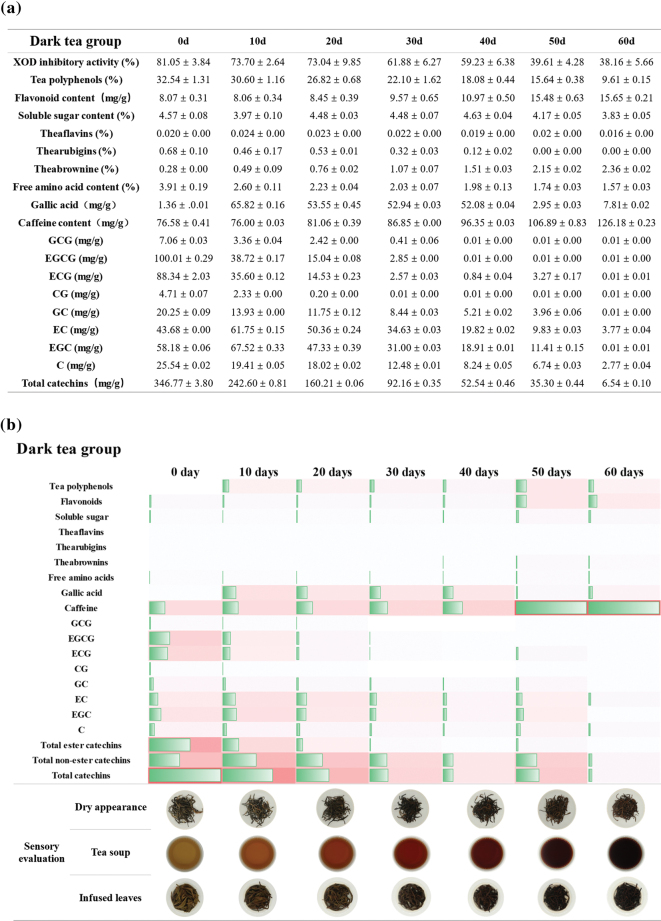
Biochemical components and sensory evaluation of dark tea with different degrees of pile fermentation. (a) The content of biochemical components in dark tea fermented for 0, 10, 20, 30, 40, 50, and 60 days. (b) 1) Heat map showing changes in biochemical components and sensory evaluation. The length of the rectangle and color intensities indicate the content. 2) The results of sensory evaluation in dark tea with different fermentation times. As the degree of fermentation deepens, the color of the dry tea, the brewed tea soup, and the brewed tea leaves were gradually deepen.

**Fig. 5:**
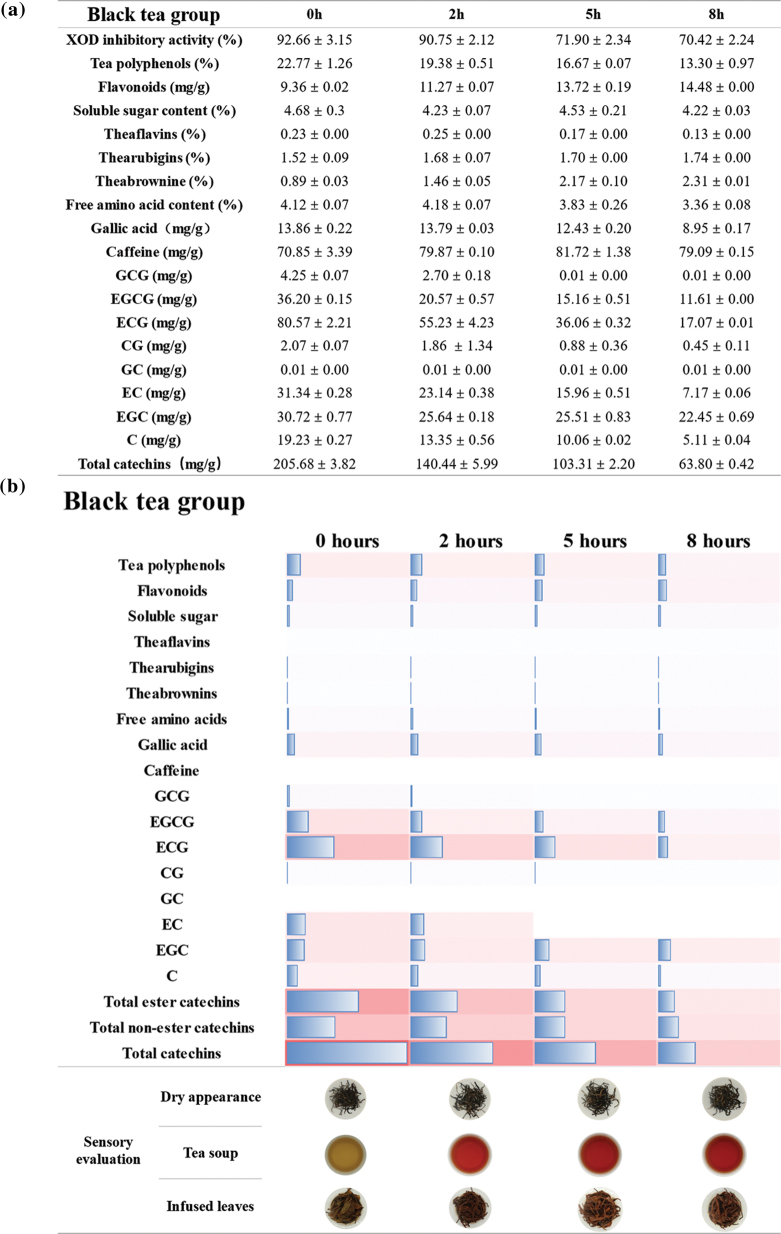
Biochemical components and sensory evaluation of the black tea with different extents of the fermentation. (a) Biochemical components in black tea fermented for 0, 2, 5, and 8 h. (b) 1) Heat map showing changes in biochemical components and sensory evaluation. The length of the rectangle and color intensities indicate the content. 2) The results of sensory evaluation in black tea with different fermentation times. As the degree of fermentation deepens, the color of the dry tea, the brewed tea soup, and the brewed tea leaves were gradually deepen.

Since the biological effects of different types of tea may depend on their composition, we analyzed the relative content of various bioactive compounds, including flavonoids, soluble sugars, free amino acids, gallic acid, caffeine, tea polyphenols, and catechins, in the tea samples. In addition, the contents of theaflavins, thearubigins, and theabrownins were specially measured in black tea and dark tea samples fermented for varying duration. The content of tea polyphenols, free amino acids, theaflavins, and catechins decreased with longer fermentation, whereas that of flavonoids, caffeine, and theabrownins showed an increase. In addition, the duration of fermentation was positively correlated with the thearubigins’s content in black tea, and an inverse correlation was seen in the dark tea. The results showed that these compounds may limit the inhibitory activity of unfermented and heavily fermented tea on urate-producing enzymes (Figures 3–5).

**Fig. 6:**
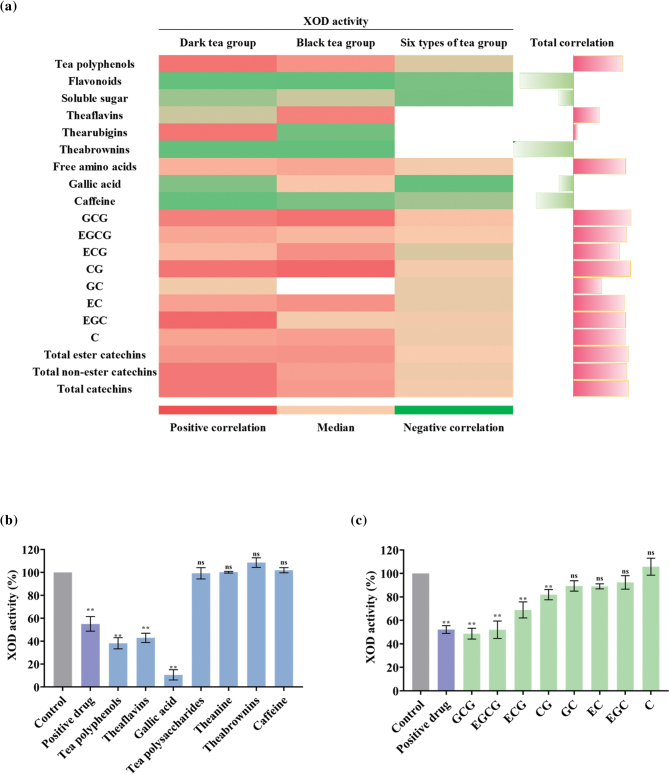
XOD activity of biochemical components and correlation analysis with chemical properties of tea. (a) Correlation between the content of bioactive compounds and XOD activity. The length of the rectangle indicates degree of correlation; red – positive correlation, buff – medium, and green – negative correlation. Positive control drug: allopurinol. (b) XOD activity in the presence of tea polyphenols, theaflavins, gallic acid, tea polysaccharides, theanine, theabrownins, and caffeine; (c) XOD activity in the presence of catechin monomers.

To further analyze the relationship between bioactive compounds and the XOD activity of tea, we analyzed the correlation between them in three groups. The results showed that the content of tea polyphenols (including catechins) and free amino acids were significantly positively correlated with the change trend of XOD activity as the degree of fermentation increases, followed by gallic acid and soluble sugar’s (Fig. 6a). In addition, the content of caffeine, theabrownins, and flavonoids was significantly negatively correlated with the inhibitory activity of XOD. Furthermore, the correlation between the changes in the contents of the eight catechin monomers in the tea after different fermentation processes and the XOD inhibitory activity of the corresponding tea samples showed that all catechin monomers in tea had a significant positive correlation (Fig. 6a).

On the basis of correlation analysis, we carried out further test with the double enzyme coupling method to verify the influence of these components on XOD activity, and the results demonstrated that bioactive group (tea polyphenols, theaflavins, gallic acid, tea polysaccharides, L-theanine, theabrownins, and caffeine) was basically consistent with the result of the correlation analysis: tea polyphenols, theaflavins, and gallic acid had a significant effect on inhibiting the XOD activity (Fig. 6b). Eight catechin monomers showed a positive correlation with XOD inhibition (Fig. 6a), of which seven (excluding C) had a significant inhibitory effect, and GCG and EGCG were the most potent. In general, the ester catechins showed a stronger inhibitory effect compared to the simple catechins (Fig. 6c).

### Tea water extracts and bioactive compounds inhibited uric acid production in hyperuricemia hepatocytes

**Fig. 7:**
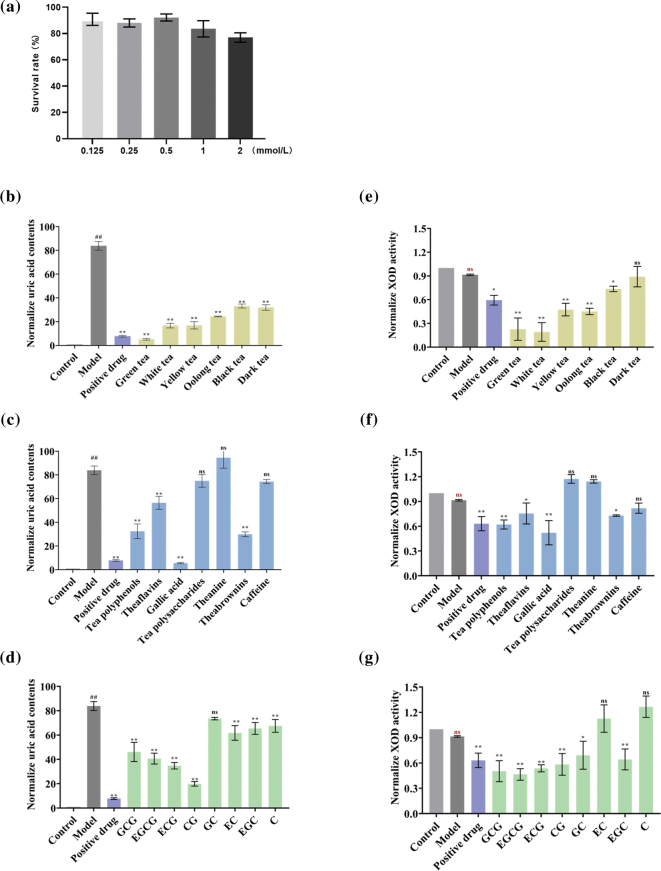
Detection of uric acid content and XOD activity of the sample-treated hyperuric acid cells. (a) L-02 cells were treated with adenosine in graded doses for 24 h. The survival rate was measured by MTT assay. (b) Uric acid content in cells after the interference of six major tea. (c) Uric acid content in cells after the interference by tea polyphenols, theaflavins, gallic acid, tea polysaccharides theanine, theabrownins, and caffeine. (d) Uric acid content in cells after the interference by 8 catechins (GCG, EGCG, ECG, CG, GC, EC, EGC, and C). (e) XOD activity in cells after interference by six major teas. (f) XOD activity in cells after interference by tea polyphenols, theaflavins, gallic acid, tea polysaccharides, theanine, theabrownins, and caffeine. (g) XOD activity in cells after interference by 8 catechins (GCG, EGCG, ECG, CG, GC, EC, EGC, and C). The data are expressed as mean ± SD. ^####^*P* < 0.0001 and ^ns^*P* > 0.1 were compared with the model, and ^ns^*P* > 0.1, *P < 0.1, **P < 0.01, ***P < 0.001, and ****P < 0.0001 were compared with the control.

Given the inconsistency between correlation analysis and enzyme activity, we evaluated the effects of different teas and their active ingredients on a hepatocyte model of adenosine-induced hyperuricemia. As shown in Fig. 7, we first determined that a 1 mmol/L adenosine was used to establish a cell model of hyperuric acid production in L-02 cells by MTT (Fig. 7a). Acting tea and its main bioactive components on hyperuricemia hepatocytes, we found that the uric acid levels were significantly lower in the culture supernatants of cells treated with the non-fermented and lightly fermented tea as opposed to the black and dark varieties (Fig. 7b). Furthermore, gallic acid showed the strongest inhibitory effect against uric acid production, followed by tea polyphenols, theabrownins, theaflavins, tea polysaccharides, caffeine, and L-theanine (Fig. 7c). Among the catechin monomers, the ester catechins were more effective compared to the simple catechins (Fig. 7e).

### Detection of tea and its main active components on XOD activity of high uric acid production cells

To confirm whether XOD was the key enzyme that inhibits the production of uric acid of tea and its components, we performed XOD activity detection on the lysis of the hyperuric acid model cells after sample treatment. The results showed that after the intervention of tea and its bioactive compounds, the intracellular XOD activity was obviously inhibited: 1) as the fermentation time increased, the inhibitory effect of tea on the intracellular XOD activity became weaker; 2) in the main bioactive components in tea, the inhibition of XOD activity is as follows: gallic acid > tea polyphenols > theaflavins > theabrownins > caffeine > L-theanine > tea polysaccharides; 3) among 8 catechin monomers, the inhibition of XOD activity is as follows: ester catechins > simple catechins (Fig. 7e–g).

### Effect of tea and its components on the mRNA expression level of XDH at the hyperuric acid L-02 cell level

**Fig. 8:**
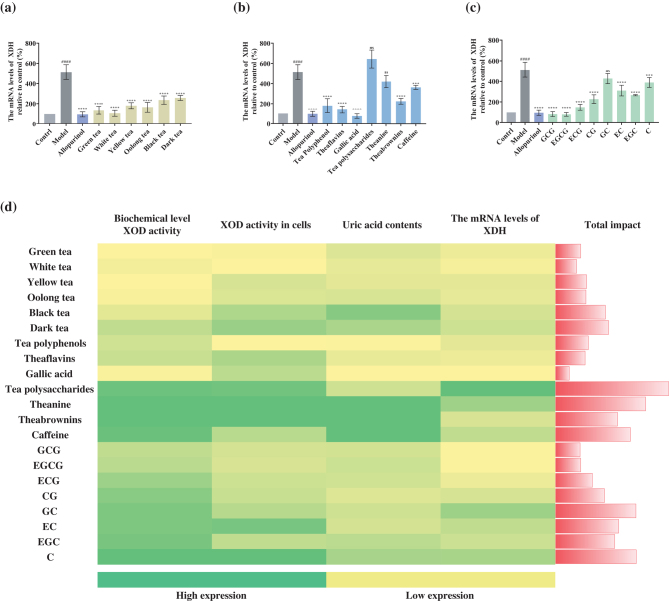
The expression level of XDH in the sample-treated hyperuric acid cells. (a) The mRNA expression level in cells after the interference of six major teas. (b) The mRNA expression level of XDH in cells after the interference by tea polyphenols, theaflavins, gallic acid, tea polysaccharides theanine, theabrownins, and caffeine. (c) The mRNA expression level of XDH in cells after interference by 8 catechins (GCG, EGCG, ECG, CG, GC, EC, EGC, and C). (d) Correlation analysis between samples (six types of tea, tea polyphenols, theaflavins, gallic acid, tea polysaccharides and theanine, and 8 catechin monomers) and the corresponding biochemical XOD activity, cellular uric acid content, cellular XOD activity, and mRNA expression level of XDH after interference. The yellower the color, the better the effect of inhibiting the production of uric acid; on the contrary, the greener the color, the worse the effect of inhibiting the production of uric acid. The bar graph on the far right represents the total effect of the samples in lowering uric acid. The shorter the rectangle, the stronger the lowering effect of the sample. Positive drug: allopurinol. The data are expressed as mean ± SD. ^####^*P* < 0.0001 was compared with the model; ^ns^*P* > 0.1, *P < 0.1, **P < 0.01, ***P < 0.001, and ****P < 0.0001 were compared with the control.

Consistent with their effects on uric acid levels, different tea extracts and its components inhibited the expression levels of XDH gene in the hyperuric hepatocytes. Non-fermented and lightly fermented tea showed a stronger inhibitory effect on the mRNA expression level of XDH. Furthermore, gallic acid was the most effective compound, followed by tea polyphenols, theaflavins, theabrownins, caffeine, L-theanine, and tea polysaccharides, as were the ester catechins compared to simple catechins (Fig. 8a–c).

## Discussion

Tea was the world’s second largest consumed beverage after water, which was made from *Camellia sinensis* (1). Various active ingredients made tea rich in health benefits, such as gallic acid had the properties of anti-inflammatory (22), L-theanine was helpful to promote mental health (23), tea polysaccharides had the potential to reduce the risk of type 2 diabetes (24), theabrownins had the properties of modulating the metabolism of gut microbiota and bile acid (25), and tea polyphenols could effectively lower uric acid (26). Abnormal uric acid metabolism may cause kidney disease, gout, and hyperuricemia. Reducing the production of uric acid by inhibiting XOD activity was an important way to treat uric acid disorder (27), which had been studied in various materials, including longan seed extract, baicalein, and gallic acid (28–30).

Generally, the researches on uric acid-lowering effects of tea had focused on either a specific type of tea (mainly green tea) or a specific type of compound (mainly tea polyphenols and catechins). For example, Chen et al. (10) found that the polyphenols in green tea significantly reduced uric acid levels, and Jung et al. (31) demonstrated that green tea extract reversed hyperuricemia. Nevertheless, the uric acid-lowering effects of various tea made with different processing, and its components had not been systematically compared yet (32, 33). Therefore, in our study, we conducted a more comprehensive analysis of the effect of tea and its main bioactive compounds on reducing uric acid by inhibiting the production of uric acid at the biochemical and cellular levels.

For tea water extracts, using traditional six types of tea made from a single variety of Yinghong No. 9, we analyzed its effect on inhibiting the production of uric acid from the biochemical and cellular levels by targeting XOD. The results showed that all the six types of tea had a significant impact on it. Even the weakest dark tea also was similar to that of the XOD inhibitor allopurinol (34). Furthermore, we came to the conclusion that the lower the degree of fermentation, the better the effect of inhibiting the production of uric acid. In other words, the effect of green tea on inhibiting XOD activity was significantly higher than that of black tea also the other kinds of tea. Inconsistent with our results, Chuang et al. (3) reported that green tea extract had a slight uric-acid-lowering effect, whereas black tea significantly inhibited uric acid production (at the dose of 2 g/kg) in the kunming hyperuricemic mice. This may be caused by the following factors. First, the raw materials of black tea and green tea used in their research may come from different varieties, which leads to inconsistencies with our results. In our research, our samples are all made from Yinghong No. 9 tea plant varieties, and the same raw materials were used to make our conclusions more comparable. Second, in order to further verify the relationship between the effect of tea extract in inhibiting the production of uric acid and fermentation, we analyzed the effect of black tea and dark tea with different fermentation times on inhibiting uric acid production. In general, from the abundance of samples and the selection of single raw materials, our results may be more reliable.

For bioactive compounds in tea, accumulating evidence reported that the content of caffeine, theabrownins, and flavonoids was higher in the heavily fermented versus unfermented and/or lightly fermented teas from the same species, whereas total catechins are present at higher levels in the latter (4, 18). This was basically consistent with the law that the bioactive compounds reduced the production of uric acid in this paper. Then, we analyzed the effect of bioactive compounds on inhibiting the production of uric acid from the biochemical and cellular levels by targeting XOD. The results showed that gallic acid, tea polyphenols, and theaflavins had a significant effect on inhibiting the XOD activity, whereas tea polysaccharides, L-theanine, and caffeine had almost no effect on it. In particular, although theabrownins had no effect on inhibiting the XOD activity in the tube experiment, it had a significant effect on inhibiting XOD at the cellular level. According to the current research, tea polyphenols and theaflavins showed a significant effect on reducing uric acid production, which was consistent with previous studies (10, 13, 35). Regarding amino acids, the correlation analysis between the XOD activity and the component content of samples, free amino acids showed a significant correlation. However, L-theanine had almost no inhibitory effect at the cellular level. Free amino acids were a determinant of tea quality. Deb et al. reported that the content of free amino acids in a cup of green tea or black tea is around 6% (36), and L-theanine accounts for almost 50% of the total free amino acids (37). Therefore, we suspected that L-theanine may not be the key amino acid that inhibits the XOD activity. In addition, whether caffeine can lower uric acid was still controversial. Towiwat et al. (38) had shown that decaffeinated coffee can reduce serum uric acid levels in participants with hyperuricemia, while caffeinated coffee cannot. In addition, Park et al. (39) advocated that moderate coffee intake may have a primary preventive effect on hyperuricemia and gout in men and women. In this study, caffeine seemed almost no effect on inhibiting the XOD activity. Regarding theabrownins, as mentioned above, it showed a significant effect on reducing uric acid at the cellular level. Here, we put forward the hypothesis that theabrownins may be one of the important components for the remarkable effect of dark tea. Moreover, our research revealed the uric acid-lowering effect of L-theanine, theabrownins, and tea polysaccharide originally.

It is worth mentioning that gallic acid exhibited an important role in inhibiting the production of uric acid, even better than tea polyphenols and theaflavins. That was never been discovered and compared before. Gallic acid, present in tea, strawberries, lemons, gallnuts, sumac, oak bark, and apple peels are a natural and healthy compound (14). Currently, lemon extract was verified the effect on lowering uric acid (40). In addition, bergenin, which can be extracted from *Bergenia crassifolia*, has the same 3,4,5-trihydroxybenzoic acid structure as gallic acid, which was also demonstrated as a novel therapeutic strategy for hyperuricemia. Hence, gallic acid may have great potential for the development of uric acid-lowering effects in the future.

For catechins, which are high in tea and are widely recognized as functional ingredients, we found that eight catechin monomers (excluding C) had a significant inhibitory effect, and GCG and EGCG were the most potent. In general, we found that the ester catechins showed a stronger inhibitory effect compared to the simple catechins. Although the EGCG content in tea was higher than GCG (41), they were epimers that can undergo interconversion under certain conditions, which explained similar inhibitory effects on XOD activity. Due to the low content of GCG, its effect is usually overlooked. But GCG had a better stability than EGCG (42). In this study, the effect of GCG on inhibiting uric acid production was basically the same as that of EGCG. Therefore, the research on the uric acid-lowering effect of GCG was meaningful, and our findings provided a theoretical basis for further investigation on its potential role in lowering uric acid levels.

## Conclusion

We established a high uric acid producing cell model by using human liver cells and compared all kinds of tea and its bioactive components on stabilizing uric acid synthesis by analyzing uric acid levels, mRNA level of XDH, and XOD activity. It was the first research to simultaneously analyze and compare the effects of polyphenols, theaflavins gallic acid, theabrownins, L-theanine, caffeine, and tea polysaccharides on uric acid metabolism. The uric acid-lowering effect of tea was very likely to be related to the degree of fermentation and the content of tea polyphenols and theaflavins. Interestingly, we found that gallic acid had the most significant effect among all the samples (Fig. 8d). This may provide a major scientific research basis for further research on the uric acid-lowering components of tea. Our study had a great guiding significance for the deep processing of tea and provided a strong scientific basis for the purchase of tea for patients with high uric acid. In addition, it also provided an important theoretical basis for the follow-up study of the regulation (synthesis and excretion) of uric acid metabolism by tea.
